# Consensus and dissent between climate activists and oil and gas employees in the United Kingdom

**DOI:** 10.1016/j.isci.2025.114229

**Published:** 2025-12-17

**Authors:** Ella Exley, Krista Halttunen, Iain Staffell

**Affiliations:** 1 Centre for Environmental Policy, Imperial College London, London SW7 1NE, UK

**Keywords:** Earth sciences, Climatology, social sciences, research methodology social sciences

## Abstract

With global temperatures breaching 1.5°C above pre-industrial levels, the energy system must undergo rapid, radical transformation to avoid irreversible ecosystem damage. This complex challenge is exacerbated by polarized views between activists advocating for a rapid end to fossil fuels and oil and gas (O&G) companies profiting from their continued use. This study examines these contrasting perspectives through ten semi-structured interviews with members of two prominent climate activist groups and employees of two international oil companies. This initial exploration identifies that activists and O&G employees agreed on the urgency of climate change, the need to reduce energy demand, the central role of government and policy, and the continued use of existing O&G projects. However, they disagreed on the scale and pace of change achievable, and the role of the O&G industry within the energy transition. The identified agreement areas suggest common ground between these groups, offering potential routes to reducing polarization.

## Highlights

• Rare juxtaposition of UK climate activist and oil & gas employee perspectives
• Common misperceptions of activist demands sustain polarized climate narratives
• Conflict-resolution tools are applied to interpret contrasting views
• An initial exploration, which calls for larger, cross-country replication

## Graphical abstract

**Figure:**
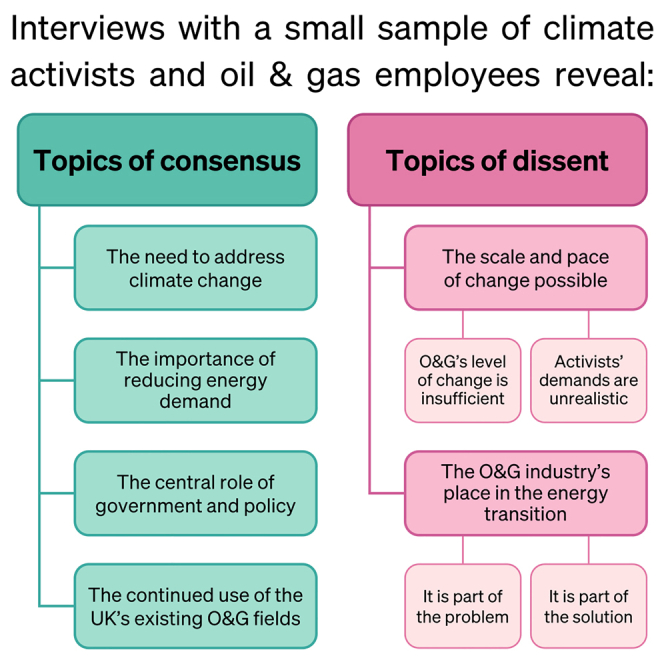

## Introduction

The energy system is the largest source of anthropogenic greenhouse gas (GHG) emissions,^1^ and limiting global warming to 1.5°C–2°C requires annual CO_2_ emissions from energy to reduce rapidly from 37 Gt to net zero by mid-century.^2^ Failing this, near-term emissions overshoot must be countered by deploying expensive negative emissions later in the century.^3^ Climate change is already intensifying heat, flood and drought extremes worldwide.^4^ In response, climate activism has globalized, from school strikes to Indigenous and community land defense, mobilizing millions of people and shaping agendas.^5^

The emotional nature of climate issues and the information-rich modern world leads to polarized views on the energy transition.^6^^,^^7^ Polarizing forces include social media,^8^ internal beliefs and values,^9^ social norms,^10^^,^^11^ and the wider cultural and political landscape.^12^^,^^13^ Differences in opinion are also driven by first-hand experience of the sector and levels of climate and energy literacy.^14^^,^^15^

Activist groups such as Extinction Rebellion and Just Stop Oil call for a rapid fossil fuel phase-out. In contrast, major oil and gas (O&G) companies continue to expand fossil fuel extraction,^16^ and remain far from net zero goals over the next thirty years.^17^ This misalignment risks serious consequences for the climate.^18^ In the UK, 75% of people are worried about climate change.^19^ Do these concerns resonate with O&G employees? What drives the disconnect between climate activists and O&G workers? Is it purely about making profits, as some activists suggest,^20^ or is the reality more nuanced?

We interview members of two prominent climate activist groups founded in the UK, and employees at two major oil and gas companies headquartered in the UK. As a pilot study of “hard-to-access” stakeholders, our sample is small (*n* = 10) and geographically constrained to the UK, so we do not claim statistical generalizability. The organizations all have global operations; however, cross-national differences in social and political context influence strategies, public support, and state responses. In the comparatively liberal UK setting,^21^ activists may face lesser consequences for protest than in more authoritarian countries, thus influencing their approaches and perceptions. Similarly, European O&G firms place greater emphasis on environmental activities than others,^22^ which may influence their employees’ perspectives. O&G employees in lower-income countries may view the energy transition differently due to development needs and oil income reliance.

This study questions how climate activists and O&G employees view the energy transition and assesses whether there are areas of agreement. It considers the discrepancy between the demands of climate activists and current positions of O&G employees, using interviews with individuals from both groups to explore shared concerns and areas of disagreement, and thematic analysis of deeper motivations behind their stances. We interpret their implications for policymakers and regulators, whose decisions critically shape corporate behavior and consumer demand. We also analyze whether these groups can learn from each other’s perspectives, questioning whether potential for reconciliation exists, as polarization can impede progress and delay solutions to urgent issues.^23^

### Polarized narratives on the mitigation gap

The energy system is the largest source of anthropogenic GHG emissions, with fossil fuels accounting for over 80% of global energy demand.^24^ Meeting the Paris Agreement goal of limiting global warming to 1.5°C requires rapid and unprecedented transformations, including a 95% reduction in coal use and a 60% reduction in oil consumption by 2050^4^ (see supplemental information for further detail). Yet, current global policies place the world on track for a 2.6°C–3.1°C rise.^25^

The energy transition (shifting from fossil fuels to clean energy sources) is frequently framed as involving starkly polarized perspectives between climate activists and the O&G sector, which stems from differing values, asymmetric information shaped by fossil-fuel lobbying and echo-chambers, and conflicting motivations around profit, risk, and climate justice.^8^^,^^26^ Groups such as Extinction Rebellion (XR) and Just Stop Oil (JSO) advocate for a rapid end to fossil fuels, calling for net zero emissions within a decade.^27^^,^^28^ XR employs non-violent civil disobedience to pressure governments, while JSO focuses on disrupting high-visibility events (see supplemental information for further description). JSO ceased UK operations in April 2025 (after this study was concluded) since the UK government ceased to issue new exploration licenses, but it continues operations in other countries.^29^ Both groups face criticism for their tactics^30^^,^^31^ but have been credited with raising public awareness.^32^^,^^33^

**Figure 1:**
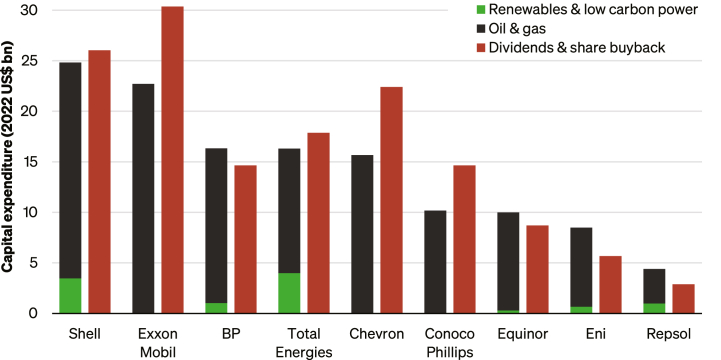
Capital expenditure of the nine largest energy majors in Europe and the US Expenditure from 2022 is split by energy type (left-hand bars) and financing activities (right-hand bars). Data from Reclaim Finance,^41^ with values from Repsol and Eni converted from Euros using USD 1.0528 = EUR 1. Oil & gas operations encompass all activities not classified as low carbon.

Conversely, major O&G companies acknowledge the need for decarbonization, but corporate strategies remain tied to expanding fossil fuel production.^34^ This misalignment reflects broader factors, as corporate strategies are shaped by policy signals,^35^ investor preferences,^36^ alongside near-term energy security^37^ and energy affordability concerns.^38^ Without strong policies to reduce fossil fuel demand, O&G companies face little incentive to reduce supply.^39^^,^^40^ In 2022, European energy majors allocated less than 25% of their capital expenditure to low-carbon projects despite record profits (Figure 1). More recent global data indicate that while clean energy investment by O&G companies is growing, it remains below 4% of their total capital spending worldwide.^42^ Critics argue that their commitments, such as reducing oil production by 20–30% by 2030, fall short of 1.5°C pathways.^17^ The renewable energy sector could see substantial job growth under rapid decarbonization, while fossil fuel jobs decline.^43^^,^^44^ Further background on the energy transition, decarbonization, and denialism within O&G companies, and climate activism in the UK, is provided in the supplemental information.

### Contrasting industry and activist perspectives

A substantial literature interrogates the corporate communications from carbon-intensive firms such as O&G companies and their lobbying.^45^^,^^46^ By contrast, studies that elicit and juxtapose the direct views of industry employees and their critics are rarer. O&G employee attitudes have been surveyed in the areas of safety-culture and risk-perception, highlighting strong professional identities and leadership influences on behavior.^47^^,^^48^ Corporate priorities and individual employee views are shown to align around corporate social responsibility issues, which can affect engagement and discretionary safety behaviors.^49^^,^^50^

Work exploring the views of industries and their critics can be found in other contentious domains, for example on wind farm siting (contrasting developers and opponents),^51^^,^^52^ the use of bee-harming pesticides (farmers, consumers, and regulators)^53^ or the taxation of sugary drinks (industry and public-health advocates).^54^ Across these settings, disagreements focus around risk perceptions, legitimacy and trust, while convergence centers on the need for state steering. Within the climate and energy sphere, national citizens’ climate assemblies have shown how deliberative processes can strengthen policy legitimacy when controversies are highly polarized.^55^

Building on the Halttunen’s exploration of O&G employees’ views on the conflict between the industry’s business model and climate change action,^56^ this study contrasts insights from O&G employees with those from climate activists, mapping both common ground and disagreements to clarify where policy can reduce polarization and build durable legitimacy.

## Results

This study explores perspectives from two distinct communities that are often cast as holding contrasting perspectives, using semi-structured interviews with members of two prominent climate activist groups and employees of two major O&G companies (Table 1). Interviews were conducted via Microsoft Teams in July and August 2023, using questions that were informed by the literature review (see supplemental information) and tailored for each group to guide the discussions (see Table 2). The motivations, values and perceptions of each group are explored using quotes from the interviews, and additional quotes are provided in the supplemental information. Quotes are labeled with the codes given in Table 1, with numbers omitted (A×, O×, OA×) where further anonymization reduced the risk of identification. Interviewees were recruited in a personal capacity only, and did not speak on behalf of, nor represent, their employers or organizations.

**Table 1:** Interview participants and their demographics

Code	Group	Gender	Age	Role
A1	Activist	F	20–30	Protest attendee
A2	Activist	F	60+	Group member
A3	Activist	M	20–30	Leadership
A4	Activist	F	20–30	Group member
O1	Oil	M	60+	Senior leadership∗
O2	Oil	F	40–50	Senior leadership
O3	Oil	M	30–40	Employee
O4	Oil	F	20–30	Employee
OA1	Ex-Oil/Activist	M	60+	Leadership∗/Group member
OA2	Ex-Oil/Activist	M	60+	Senior leadership∗/Leadership

Interviewee roles and the organizations they are affiliated with, are anonymized. Codes: O = oil; A = activist; OA = oil/activist; ∗ = retired.

**Table 2:** Questions used for each group of interviewees

	Questions for O&G interviews	Questions for activist interviews
1.	Can you briefly introduce yourself and your role within the O&G industry?	Can you briefly introduce yourself and your experience within climate activism?
2.	What motivated you to join the O&G industry?	What motivated you to get involved with climate activism?
3.	In general, what are your thoughts on climate activism? a. Its purpose? b. Its effectiveness?	In general, what are your thoughts on climate activism? a. Its purpose? b. Its effectiveness?
4.	Are you familiar with the demands of UK climate activist groups? a. What is your response to their demands? b. What is your response to the split opinions regarding groups such as JSO?	What is your understanding of the demands of UK climate activist groups? a. What is your response to the split opinions regarding these groups?
5.	Climate activist groups often single out O&G companies in their campaigns/actions as the “bad guys,” what is your response to the negative perceptions climate activists have toward the O&G industry? a. What is your response to the criticism that O&G companies are not decarbonising fast enough?	Do you have any opinions or comments you would like to share on the actions of O&G companies? a. Should the UK license new oil and gas projects? b. Should existing UK fields continue production, and if so for how long?
6.	Are there any areas where you believe climate activists and the O&G industry might share common ground?	Are there any areas where you believe climate activists and the O&G industry might share common ground?
7.	What is your experience of the relationship between your company and climate activists? a. Have you personally engaged with any activist groups? b. If so, were these engagements beneficial?	How would you allocate responsibility across government, the O&G industry, and consumers?
8.	Do you think a conversation between these two groups could ever be helpful?	Do you think a conversation between these two groups could ever be helpful?

**Figure 2:**
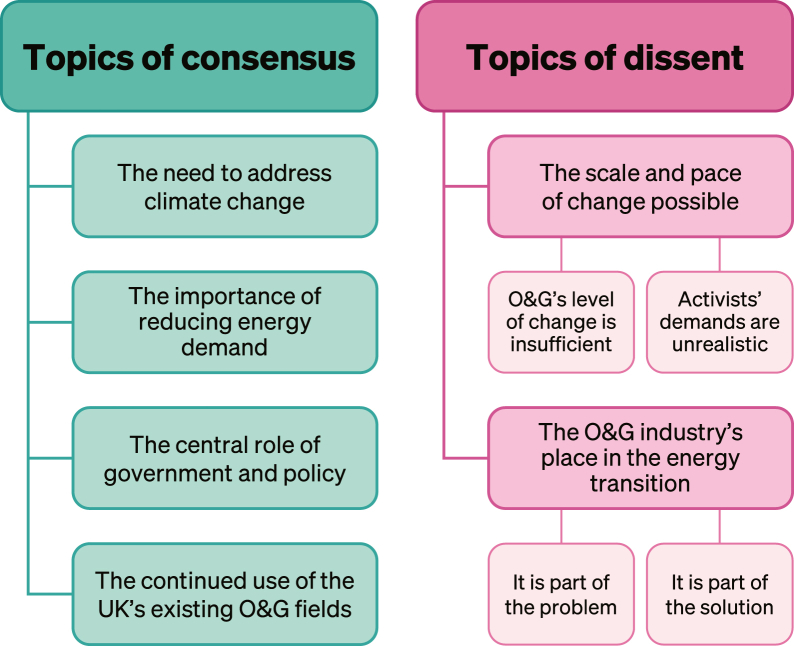
Topics of consensus and dissent Topics identified through analysis of interview responses between climate activists and O&G employees in the UK.

Analysis of these reveals that despite being portrayed as having diametrically opposing aims, there are four areas of shared values alongside two areas of disagreement (Figure 2). Given the limited number of participants, these findings are presented as exploratory, providing initial insights into areas of consensus and dissent, which should be expanded upon with larger and cross-country samples.

### Areas of consensus

Despite strong disagreements, four previously unacknowledged areas of alignment related to the energy transition were found in our interviews, around the need to address climate change, the importance of reducing energy demand, the central role of government in managing the transition, and the continued use of existing reserves. Identifying and focusing on these could improve the productivity of future dialogue.^57^

#### The critical need to address climate change

All climate activists displayed considerable concern for climate change, with several expressing fear. A2 vividly described the world as “*literally burning,”* with humans and animals “*cooking to death.”* A3 felt “*absolutely terrified”* that climate change is “*time limited”* and set to get “*exponentially worse.”* Most activists believed the O&G industry lacks concern for climate change, citing examples of “*rampant denialism”* (A4). Some referenced industry denial and delay strategies, similar to those identified in the literature.^58^^,^^59^ A1 noted that Shell “*raised their oil platforms to account for sea level rise while also publicly denying that sea level rise is going to happen,”* as documented by the New York Times^60^ during a period when Shell downplayed climate risks.^61^ A3 mentioned BP’s “*personal carbon footprint calculators”* as an attempt to shift emissions responsibility from producers to consumers.^62^ On a personal level, OA2 referred to former colleagues, stating “*they are in denial.”*

Although activists view the O&G industry as “*absolutely denying”* (OA2) climate change, all interviewed O&G employees expressed personal concern for global warming and agreed “*something needs to be done”* (O2, O3). Their discussions of climate change were less emotionally intense, focusing more on scientific information than personal fear. For instance, O1 supported the Paris target of “*trying to stay within 1.5°C”* and stressed the urgency of action based on the diminishing “*available carbon budget.”* Most O&G interviewees acknowledged past industry denialism but expressed confidence in a shifting perspective. O3 stated “*no one’s denying this at all, we know the numbers,”* while O4 noted “*no one’s a climate denier”* and “*everyone agrees on the importance of decarbonizing.”*

Their views align with the public stances of some European oil companies, for example, endorsing the Paris Agreement.^63^^,^^64^ However, this does not prove that “deniers” no longer exist within the O&G industry. OA1 referenced an industry survey showing high acceptance of climate change, but noted that “*not all companies are the same,”* with European companies considered more progressive than American ones, for example.^65^

#### The importance of reducing energy demand

A second consensus topic is the need to reduce energy demand for effective decarbonization. Demand reduction’s role in limiting global warming is widely recognized.^66^ The IPCC expects steep demand reductions in its 1.5°C pathways,^4^ and both interviewed groups agreed on this aspect.

Activist interviewees understood the need to decrease energy demand, anticipating that the “*quality of life will change”* (A4). Some have adopted this personally, such as by avoiding air travel and meat (A2 and OA2). A2 expanded on distancing themselves from an unsustainable “*capitalistic mindset,”* “*not buying things,”* and “*learning to grow food.”* A2 invested heavily in retrofitting their home with a heat pump and solar panels. Despite individual efforts, activists view O&G companies as “*absolutely opposed”* to demand reduction as it threatens “*their business model”* (A3). OA2 claimed “*demand management is very, very unpopular”* within the O&G industry, a view that activists reinforce, characterizing the industry’s “*agenda”* as “*feeding the world more oil and gas.”*.^20^

All O&G interviewees acknowledged the need to reduce hydrocarbon and overall energy demand. O1 warned that “*stopping production before demand has been reduced is problematic”* because society “*won’t be able to run”* given the heavy dependence on oil and gas for electricity, transport, and heat. O3 similarly noted that currently “*nothing is in place to make this a reality.”* Reflecting on global O&G dependency, most employees agreed that “*we should be doing everything we can to reduce demand quickly”* to enable reductions in production (O1). O2 emphasized that achieving the Paris Agreement is unrealistic “*if everybody consumes energy the way that the West has over the last couple of decades.”* Employees recognized that demand reduction and transitioning to renewables will require adjustments in the “*quality of life”* (O3); however, none disclosed their own lifestyle changes.

While this alignment with climate activists on demand reduction is noteworthy, corporate views differ. For example, BP^67^ and Shell^68^ have partially acknowledged the need to reduce hydrocarbon demand, committing to lower oil production by 20–30% and 10–20%, respectively, between 2019 and 2030. Shell met its target in 2023 but is not pursuing further reductions.^69^ Both companies emphasize emissions reductions over demand reduction,^67^^,^^68^ raising questions about O&G companies’ commitment to decreasing hydrocarbon demand. Long-term scenarios, such as BP Net Zero and Shell Sky, limit warming to 1.5°C–2°C through systemic policy measures and technological change rather than unilateral corporate production cuts, highlighting the limited influence of any single firm in the global oil market.^70^^,^^71^

#### The central role of government and policy

A third point of consensus is the government’s role in steering the energy transition. O&G employees recognized that government regulations set the pace of change, with OA1 stating that O&G companies “*can only move as fast as the external government.”* Climate activists have criticized O&G for presenting itself as a “*passive player”* constrained by other stakeholders such as governments or consumers (OA2). However, all interviewed activists agreed that the government bears “*responsibility”* due to its control over the economy (A3). A3 further explained that [climate activists] deliberately direct “*the framing and the messaging and even the demand”* toward the government, despite identifying the fossil fuel industry as its primary adversary. Activists view the government as key to regulating and addressing the issue.^72^

This suggests potential collaboration between climate activists and O&G employees through policy advocacy, with mutual support for stronger regulations toward change. OA1 suggested that O&G companies should lobby alongside activists for demand-reduction policies, such as “*heat pump installation,”* to demonstrate “*they are serious”* about the transition. This aligns with the concept of “boundaryless activism”^73^; however, the feasibility of joint advocacy is unclear. Several activists mentioned that O&G companies already have “*huge policy impact”* (A1) and pay to “*slow down change”* (OA2). While the extent of their influence is unknown, InfluenceMap estimated that the five largest O&G firms spend $200 million annually on lobbying to delay or block climate-related policies.^74^ This imbalance of power poses a substantial challenge to collaboration.

#### The continued extraction of oil and gas in the UK

An unexpected topic of agreement in the interviews was that current UK oil and gas projects should continue to be used while demand remains high. All O&G interviewees thought this was sensible, drawing on economic, political, and environmental considerations. For instance, O1 explained that “*from the point of view of sound national economic and security supply,”* the UK should produce O&G “*from its own resources”* rather than “*send dollars abroad.”* O1 added that, in “*net terms,”* the “*CO*_*2*_ *from imported oil could potentially be higher”* than oil produced domestically once “*production and transport”* have been accounted for. The UK’s domestic oil and gas production has lower carbon intensities than imported fuels, except for those from neighboring Norway.^75^^,^^76^

All O&G interviewees believed JSO activists advocate for an immediate halt to oil production. OA× claimed “*most of them instinctively feel that shutting oil production down will solve everything.”* O3 said that JSO activists believe “*we can just move over, just give up the hydrocarbons”* when it is “*not that simple.”* Similarly, O4 suggested JSO want O&G companies to “*just turn off their fields,”* adding that “*they’re not going to.”*

This misinterprets JSO’s demands. Interviewees acknowledged this stance is often misunderstood: “*a lot of people think [JSO are] naive to the fact that if we just turn oil off, chaos would go down.”* Their demand is “*no new oil and gas, not stopping now,”* as stated on the JSO’s website.^27^ Both groups therefore agree that UK O&G production should continue in the near term, despite O&G employees anticipating disagreement. Journalists also misinterpret JSO’s messaging, focusing solely on their name: “*the biggest single problem with Just Stop Oil is that there is no just stopping oil.”*^77^ This widespread misunderstanding suggests JSO might reconsider their communication approach. Many overlook the subtleties of JSO’s stance, including OA×, who is “*aware of the headlines”* but has not “*read their prospectus.”* XR’s demands face similar misinterpretation, as discussed in The scale and pace of change possible. XR activists have vocally opposed new O&G projects such as the Horse Hill oil field expansion and Rosebank development.^78^

Regarding the duration of continued O&G production, interviewees referenced a common talking point that the UK has a reserves to production ratio of less than 8 years,^24^ suggesting limited scope for future production. A3 stated “*if we treat this like the emergency it is, we can fully transition in that time.”* OA2 emphasized the importance of the UK government not granting new licenses for exploration to expand supply. This aligns with recommendations from the IPCC,^4^ the IEA,^79^ and the Climate Change Committee,^80^ as well as the campaigns of XR. It also sends an “*important message to other countries”* to follow suit (OA2). While most O&G interviewees did not comment on new licenses, O1 agreed that halting exploration in the UK is “*economically sensible,”* reasoning that new oil production would likely be “*worthless”* by the time it is available due to climate change mitigation efforts reducing demand, mirroring many industry scenarios.^81^ However, projections for the timing of this decline vary widely across energy agencies and O&G companies, exemplified by the approval of the Rosebank oil field.^82^

### Areas of dissent

Two areas of disagreement were identified from our interviews, surrounding the achievable scale and pace of change, and the O&G industry’s ability to support the transition. The aim of addressing these differences is not to establish truth but to clarify and explore the specific points of conflict. This approach creates opportunities for reframing to reduce polarization, establishing a foundation for future engagement by highlighting areas that require attention.^57^

#### The scale and pace of change possible

Interviewed activists argued that the O&G industry is resisting decarbonization due to an unwillingness to transition, indicating skepticism about the O&G interviewees’ justifications for the current pace of change. Conversely, some O&G employees criticized the net zero timelines proposed by climate activists as “*unrealistic”* (O4). Consequently, the scale and pace which the O&G industry can transition remains contested. Four key points of disagreement in this category are: whether the industry is willing to change, is doing enough to transition, has valid reasons for not moving faster, and what the overall pace of the green transition should be.

Most activists doubted the O&G industry’s willingness to decarbonize, with many questioning how much change has occurred so far. Drawing from their career at an oil major, OA2 observed, “*When I joined, [the company] was a company talking about change. When I left, it was still a company just talking about change. There has been no change.”*. Activists see this as a sign that O&G companies are “*actively choosing not to participate”* (A4). A4 highlighted continued fossil fuel investments as evidence of indifference to IEA insights,^83^ stating “*you either believe the IEA … or you don’t”* and O&G companies have “*chosen the path they want to take.”*

O&G employees disputed claims that “*there has been no change.”* OA1 argued, “*that’s not really true,”* as the level of change is often overlooked. O3 described their company’s latest strategy as “*a huge improvement”* from when they joined many years ago, and O2 noted a “*significant shift”* within the industry, claiming the Paris Agreement sparked a “*race to the top.”* They argued that characterizing the industry’s perceived lack of transformation overlooks the complex factors that influence industry decisions and actions. For instance, O3 stated, “*it’s not that we don’t care,”* “*we know the numbers [and] believe [them].”* Rather, employees emphasized the need to balance climate goals with a wide array of considerations. OA1 claimed, “*there are a whole bunch of practical constraints which are stopping the speed at which we do this rather than anything in principle.”*

Increased investments in renewables by O&G companies were cited as evidence of change, with OA1 noting that oil companies are investing substantially more than a few years ago. While this challenges OA2’s assertion and suggests some change, the proportion of CapEx allocated to renewables by leading O&G companies has been described as inadequate^41^ (see Figure 1). Pathways compatible with 1.5°C targets require rapid sector-wide reductions in oil and gas production (3% per year), implying no new oil and gas fields.^84^^,^^85^ The O&G sector’s production plans are not aligned with 1.5°C targets,^17^^,^^86^^,^^87^ with only a small minority of companies close when assessed against absolute production budgets.^88^ Thus, OA2’s claim of “*no change”* is not robust, nor is the position that sufficient change has been made, although no O&G interviewees explicitly endorsed such a stance. O2 characterized the industry’s shift as “*significant,”* yet also agreed “*not enough is happening.”*

O&G interviewees attributed the energy transition slowdown to two main factors: energy security and shareholder influence. Most highlighted government pressure for affordable energy, with OA1 noting this intensified amid energy security concerns from the Russia-Ukraine war. O4 explained, “*people being unable to afford their energy bills is a much shorter-term problem than climate change,”* and this “*year-to-year”* issue demands attention. An estimated 4,000 UK deaths resulted from living in cold homes due to unaffordable energy bills during the 2021-22 winter.^89^ These interviewee accounts map directly onto the broader drivers of O&G decision making outlined in the literature: that policy, energy security, and affordability post-2022 shape corporate choices,^35^^,^^37^^,^^38^ and in turn employee perceptions.

The interviewed activists rejected the dilemma between affordable energy and the transition to renewables. A2 and A4 both perceive the war in Ukraine as “*a blessing”* (A2) for O&G companies, allowing them to continue “*extracting and exploiting fields”* while “*pretending to do good”* (A2). A4 noted this enabled them to make “*a load of money because of energy prices.”* In 2022, Shell and BP profits doubled to £32bn and £23bn, respectively,^16^ influencing Shell’s decision to halt annual production cuts^69^ and BP weakening its 2030 O&G reduction commitment from 40% to 25%.^90^

Another constraint is the need to satisfy shareholders. O3 said, “at the end of the day, people have invested in us, and we need to give them a return on their capital … that’s just what a company is.” O2 added that many investors “don’t care about climate” and expect O&G companies “should make as many returns as possible and provide that to them.” This was reflected in 2023 shareholder votes at various O&G companies, where climate resolutions were supported by only 17% of voters at BP, 20% at Shell, and 30% at TotalEnergies.^91^ Despite rising support for sustainability resolutions between 2006 and 2022, they remain a minority view.^22^ One interviewee highlighted a major company revising its O&G reduction commitment due to energy security concerns: the decision “received backlash by activists, however the share price went up 25%.”^90^

Friedman famously asserted that a company’s primary duty is to its shareholders,^36^ a doctrine that has shaped capitalism for decades. However, perspectives evolve, with growing emphasis on broader stakeholder interests, including environmental concerns. With O&G companies, a paradox persists in which wealth is created for shareholders while being destroyed for others through worsening droughts, wildfires and floods.^92^

The industry’s need to generate shareholder returns did not resonate with the interviewed activists as a reason for delaying the transition. A2 criticized O&G companies for “*dragging their feet just for money,”* while A3 condemned the human cost of “*millions and millions of people dying and being forced from their land”* due to “*corporate greed.”* A3’s comments echo the IEP’s projection that 1.2 billion people could be displaced by 2050 due to climate change,^93^ potentially resulting in many fatalities. Comparing concerns about climate refugees (A3) with unaffordable energy bills (O4) reveals a core tension within O&G companies’ inaction: how can they balance addressing the immediate problem of energy poverty (5 million deaths per year from cold exposure)^94^ against a future where millions may die from climate change? Activists’ empathy for this tension seems limited. While such dilemmas risk deadlock, Lamb et al. contend that even intractable justifications are ultimately discourses of climate delay.^45^

While activists criticized the level of change within the O&G industry, O&G employees questioned climate activist groups’ ambitious timelines for a complete transition. XR called for^28^ net zero by 2025, while JSO argued the UK “*can fully transition”* to renewables by 2030 (A×). O1 stated, “*the idea that we could stop emissions by 2025 or 2030 seems highly improbable.”* OA1 labeled these campaigns as “*misguided,”* explaining, “*you can’t decarbonise something quickly, the whole system is going to take decades.”* A1 countered that “*If you’re getting hung up on the timeline … you’re missing the point,”* as these demands express “*unrest and dissatisfaction”* with climate action rather than absolute mandates about timelines. While getting “*bogged down”* in details can be unproductive, O2 noted that “*unrealistic”* statements can make “*the conversation difficult”* and disengage O&G companies: “*if it’s not achievable, why are we expected to achieve it?.”* This suggests activist groups could refine their communication strategies to avoid misinterpretation, and that O&G should not “*read too much into”* the timelines put forward by climate activists (A×). XR’s website discussed the 2025 net zero goal, aiming to stimulate immediate action,^95^ highlighting that a 2050 target invites harmful delays and reliance on unproven negative emissions technologies.^96^

The achievable extent and pace of the energy transition remain contentious areas. Interviewed activists argued that the O&G industry’s decarbonization efforts fall short, while O&G interviewees claimed that the decarbonization pace is shaped by many factors beyond climate, such as energy security and shareholder interests. While they challenged activists’ proposed transition timelines, what the O&G industry considers realistic is falling far short of what is necessary.^86^

#### The oil and gas industry’s place within the energy transition

The O&G industry’s climate change denialism, harmful practices, subversion of the United Nations COP climate change conferences,^97^ limited decarbonization efforts, and characterization as “*the bad guys”* (O4) lead interviewed activists to not trust it to contribute constructively to the energy transition. Conversely, interviewed O&G employees believe the industry can play a positive role; many described feeling personally motivated to drive decarbonization, with some dedicating their careers to this goal. A fundamental point of contention between these groups is whether the industry is inherently part of the problem, or can become the solution as OPEC’s secretary general claims.^98^

Most activist interviewees echoed A2’s view of the O&G industry as “*the enemy,”* “*our killers,”* and “*evil.”* A1 added, “*I just don’t think they’re the good guys, and I think they’ve shown us enough evidence.”* Evidence cited by activists includes the industry’s “*rampant denialism”* (A4) and perceived reluctance to change. Some activists also criticized “*harmful oil and gas practices abroad”* (A1) such as TotalEnergies’ unjust displacement of “*tens of thousands of people”* (A3) to build the East African crude oil pipeline.^99^

Interviewed activists generally see no role for the O&G industry in the energy transition. A1 commented “*that kind of company shouldn’t hold the power it has,”* and OA2 explained that the O&G industry “*can only change by accepting that it doesn’t have a role to play.”* While O&G interviewees highlighted complexities that hinder rapid decarbonization, OA2 contended that no excuse is valid and that such explanations only reinforce the industry being “*the problem”* rather than “*part of the solution,”* due to the imperative that “*we stop using O&G.”* This is not a unique stance. Scholars argue that the industry simply cannot continue producing with no defined end, with Hoffman and Ely modeling three distinct pathways for its controlled demise.^92^ Additionally, 1,800 NGOs within the Climate Action Network are committed to revoking the social and economic licenses from fossil fuel companies,^100^ reinforcing A4’s assertion that “*a lot of people want to dismantle the fossil fuel industry.”*

Some O&G interviewees acknowledged the industry’s negative image. O4 recognized the view of the industry as “*the bad guys”* as “*not a totally wrong opinion,”* citing ExxonMobil’s cover up of scientific evidence on anthropogenic global warming in the 20^th^ century as “*horrible,”* and recognizing that “*if action had been taken at that time … we would be a lot further along in the energy transition.”* However, OA1 cautioned against oversimplification, stating “*it’s much easier to say oil companies are bad bastards and it’s all their fault”* rather than accept that it is a “*much more nuanced story.”* It could be argued that dismissing dilemmas articulated by the O&G industry (such as short-term deaths vs. long-term deaths) as part of an “*evil”* plan (A2) for delay is an easier narrative than trying to address these complex issues.

Despite awareness of being seen as “*the problem”* by activists (OA2), all interviewed O&G employees believed their industry could contribute meaningfully to the energy transition. First, they argued that the industry’s scale can expand low-carbon technologies. O3 emphasized harnessing “*the power and size”* of the company to bring “*costs down”* and “*scale up”* these technologies. O1 sees potential to excel in carbon capture and storage (CCS), given the industry’s expertise in large-scale process engineering and fluid dynamics. This is important as CCS is considered essential by the Climate Change Committee for net zero.^101^ Second, O&G interviewees expressed optimism about their role in the energy transition, driven by personal motivation to find solutions. O4 chose to join an oil major in the hope of gaining “*influence”* and “*decision-making power”* to apply their environmental education in an impactful way. O3 sees themself “*working […] to try to find solutions,”* while OA1 stated, “*I want to get stuck in and try and solve things.”*

This solution's focus on O&G interviewees contrasts sharply with activists’ portrayal of the industry as “*the problem”* (OA2). Some O&G interviewees implied their engagement with energy transition challenges reflects greater efforts than of activists. OA1 voiced frustration with an activist group, claiming they “*don’t propose any solutions at all.”* Similarly, O3 remarked that activist groups are “*very good at shouting”* but “*don’t have solutions.”* When presented with this perspective during interviews, activists responded that crafting solutions is not their responsibility. A× explained that their group had not devised a strategy for the UK government if they agree to halt new O&G licenses because “*that’s not really our job.”*

Activists view the “*change the company from within”* approach of O&G employees with skepticism,^102^ seeing it as either self-deceptive or naive. A3 commented, “*people have all sorts of ways of justifying what they’re doing if it’s putting them in a good situation.”* OA2 noted from their experience that O&G employees often believe they are “*better than the other guy,”* with a “*little lawyer”* inside their head justifying their actions. This sentiment is echoed by industry leavers. Reflecting on his departure from Shell, Quinn stated^102^ “*so many working there perform appalling mental gymnastics to convince themselves that they’re doing good things for the world”* and that those genuinely attempting transformation are on “*a fool’s errand.”*

Views on the O&G industry’s role in the energy transition reveal stark contrasts between activists and O&G employees. To enable more productive future discussion, activists could propose roadmaps or solutions, although they state this is not their role. The O&G industry could actively build trust by investing more in the transition, but may be discouraged by lower profits than fossil fuels. There are no easy solutions.

## Discussion

Climate activists and O&G companies can seem diametrically opposed. Activists oppose the continuation of fossil fuels, while O&G companies and their employees depend financially on them. Through analyzing interviews with climate activists and O&G employees (speaking in a personal capacity), this study found four areas of agreement and two points of conflict around the energy transition. Identifying shared views and the specific disagreements could support mutual learning and reduce polarization.

The first area of consensus among all interviewees, including O&G employees, is that climate change is an urgent issue. However, this does not eliminate activists’ concerns about industry denialism, especially as interviews were limited to representatives from European O&G companies seen as more progressive,^65^ and individual views may diverge from company positions. Future research could expand this work to include viewpoints from less progressive companies, such as those headquartered in the US, and compare individuals’ views with the official statements and actions by companies.

The second consensus is the need to reduce hydrocarbon and overall energy demand for decarbonization, with both groups recognizing lifestyle impacts. Despite potential conflicts with O&G business models, both groups somewhat agreed on the importance of demand reduction. Support for energy efficiency measures such as electric vehicles, heat pumps, and improving building insulation, which reduce energy consumption without compromising living standards, could gain support from both sides. This suggests specific areas where constructive efforts could be pursued. However, demand reduction runs counter to O&G business models. Further research should examine corporate stances on the energy transition, employing discourse analysis of company documents, financial reports, media coverage, and industry benchmarks.

The third consensus is the government’s central role in steering the energy transition, presenting opportunities for aligned policy advocacy. However, the feasibility of such efforts requires a profound change in O&G companies’ current policymaking influence, often used to delay or block climate action.^74^ Given that both groups view government and policy as driving the energy transition, recent developments such as the UK government’s “mission-based” approach toward the energy transition could be seen as positive by both sides. Stronger long-term commitments to decarbonization might help to satisfy both groups, provided that policy efforts are genuine and not undermined by conflicting interests. To move beyond inference, future work could capture policymakers’ and regulators’ perspectives, to clarify how institutional constraints, electoral cycles, and regulatory mandates shape transition pace and whether the areas of consensus identified here translate into implementable policy.

The final consensus is that existing O&G reserves should continue being used until depletion. This is notable as it clarifies a common misperception that JSO advocates for an immediate halt to oil production, potentially fueling unnecessary polarization. This suggests opportunities for activist groups such as JSO to refine communication strategies.

While these unexpected points of agreement could reduce misunderstandings and polarization between climate activists and the O&G industry, significant divides remain on two major topics: the scale and pace of change possible within the energy transition, and whether the industry can contribute constructively. Agreement on the transition’s scale and pace is unlikely, given the financial losses O&G companies would face by ramping down their core operations while still profitable. This aligns with the idea that motivated biases (such as protecting economic interests and national security) can influence stakeholder perspectives.^103^ Divergent views may also reflect heterogeneity in first-hand industry experience and domain knowledge about climate change, energy demand, and the energy transition (i.e., energy literacy), which influence risk perception and policy pref.^14^^,^^15^ Strong decarbonization policies are likely to be more effective than expecting businesses to forgo short-term profits for the common good; a view shared by both groups interviewed here. Although O&G employees wish to see their industry involved in the transition, its past actions lead to questions over whether a decision-making role is merited.

The researched groups remain at an impasse on these disagreements, primarily due to activists’ deep distrust of O&G companies. A productive avenue for future research could explore strategies for the industry to rebuild trust. Ultimately, areas of consensus do exist between the groups, suggesting that the disconnect may not be as entrenched as it is often portrayed. This is interesting because it contrasts with the conventional view that there is no agreement between oil companies and oil activists. Areas of agreement are often overshadowed by broader public and partisan polarization surrounding climate change, which impedes constructive dialogue.^23^ Identifying specific areas of mutual interest, such as support for energy efficiency measures or pushing governments to commit to stable, long-term decarbonization policies, might help bridge the divide and foster more constructive debate in the future.

### Limitations of the study

A key limitation of this work is the inclusion of only two climate action groups and two O&G companies, represented by ten interviews in total. This small sample size means the findings presented here should be considered as exploratory in nature and for thematic identification, not broad generalizations. While qualitative research lacks strict guidelines on sample size,^104^ ten interviewees may be considered a small number. The “elite” status of interviewees limits their availability in academic research. This aligns with recommendations to work “with greater care with a few people [rather] than more superficially with many.”^105^ Comparable studies use similar sample sizes,^106^ and samples of 6–12 participants can be “extremely valuable and represent adequate numbers” when researching “hidden or hard to access populations.”^107^

Interviewing individuals offers nuanced insights, but deriving overall conclusions about the organizations they represent is challenging, as interviewee perspectives may be shaped by personal views and biases rather than organizational stances. Further, given the small sample size and exploratory approach, not all viewpoints within each group may be fully captured, despite steps to minimize sampling bias. For example, reaching activists aged 30–60 proved challenging, despite a relatively balanced age distribution.^108^ Also, while categorizing interviewees as activists or O&G employees aids comparison, it can oversimplify realities. Two interviewees transitioned from long O&G careers to climate activism in retirement, prompting a new hybrid category: OA (See Table 1). Future research with larger and more diverse samples will be required to assess the robustness and generalizability of the themes we identify here. If policymakers or regulators were included as participants, this would improve upon our work’s ability to triangulate the decision-making context that was identified as central by interviewees.

## Resource availability

### Lead contact

Further information and requests for resources should be directed to and will be fulfilled by the Lead Contact, Iain Staffell, i.staffell@imperial.ac.uk.

### Materials availability

This study did not generate new materials.

### Data and code availability

• De-identified excerpts from the interview transcripts are provided in the article and supplemental information. Full interview transcripts cannot be shared publicly due to confidentiality restrictions. Requests for access may be considered by the authors, subject to approval by the CEP ethics panel.
• No custom code was generated.
• All other items are provided in our supplemental information.

## Acknowledgments

This work was supported by the 10.13039/501100000266Engineering and Physical Sciences Research Council [EP/R045518/1].

## Author contributions

EE: conceptualization, methodology, investigation, data curation, and writing – original draft. KH: conceptualization, supervision, and writing – review and editing. IS: conceptualization, supervision, and writing – review and editing.

## Declaration of interests

The authors declare no competing interests.

## STAR★Methods

### Key resources table

**Table:** 

REAGENT or RESOURCE	SOURCE	IDENTIFIER
**Software and algorithms**		

Teams	Microsoft	https://www.microsoft.com/en-gb/microsoft-365
Word	Microsoft	https://www.microsoft.com/en-gb/microsoft-365

### Experimental model and study participant details

#### Human participants

All interviews followed relevant institutional guidelines, and approval was received from the CEP Ethics Panel on 15 June 2023. Informed consent was obtained from all participants, who were given clear information about the study, emphasising their voluntary participation and the right to withdraw at any time without consequence. All interviews were conducted in English with participants based in the United Kingdom. No honoraria were provided. Participants gave explicit consent for publication of de-identified verbatim quotations.

Participants were anonymised and represented by identifier codes, which are listed in Table 1 with participant metadata. The sample size was *n* = 10 interviewees, which were allocated into three groups of four activists, four O&G employees, and ex-O&G employees who became activists. Demographic information was collected on age and gender (Table 1). To reduce the risk of identification in this small sample, we generalised job roles to broad categories and reported ages in ranges. Other variables such as ancestry, race/ethnicity, and socioeconomic status were not collected or analyzed, as the assessment of how these factors shape perspectives was outside the scope of the proposed study.

Interviews captured individuals’ personal views, and participants did not speak for their employers. When discussing ‘companies’, we refer to documented corporate strategies, disclosures or third-party assessments. When we discuss ‘employees’ we refer to interview data. We note that investment and portfolio decisions rest with boards and senior executives accountable to shareholders and regulators, not with most employees.

Before publication we conducted a review of the risk of re-identification for all quotations. Where needed, excerpts were paraphrased, or potentially identifying descriptors were removed (e.g., specific organisations, career paths, technologies, or locations), following best-practice guidance.^109^^,^^110^

### Method details

#### Study design

This study is exploratory and inductive, using semi-structured interviews to analyze the alignment and misalignment of views between climate activists and O&G employees. Semi-structured interviews were conducted with O&G industry professionals and climate activists. Interviews were used for their ability to capture personal perspectives and experiences, allowing for nuanced understanding of the contentious energy transition. Interviews are relatively underutilised in this field; while activists are frequently studied, insights from O&G employees are rare in climate research.^56^ A semi-structured interview format enabled inductive theory development, combining fixed questions with flexibility to explore themes introduced by interviewees.^111^ This approach is suited to capturing complex perspectives in emotion-driven discussions, where rigid questioning may inhibit authentic expression.^112^

The research focuses on members of UK-headquartered climate action groups and O&G companies for two reasons. Firstly, due to the considerable variation between different organisations worldwide, it was pragmatic to limit the geographical scope to the UK. Secondly, all four organisations have high visibility, resulting in abundant literature on their activities to corroborate interviewee quotes against.

#### Recruitment and sampling

Purposive sampling was used to ensure interviewees had relevant expertise.^113^ Activist interviewees were selected for sustained commitment via repeated event attendance or leadership roles. O&G interviewees were chosen with a minimum of five years’ industry experience in energy transition roles. Initial interviewees were identified and contacted via LinkedIn and professional networks, followed by a ‘snowball’ recruitment method where initial interviewees were asked to recommend further suitable candidates.^114^ To minimise convenience bias, interviewees represented varied roles and age groups. The exploratory nature and limited sample size (*n* = 10) mean that results should be considered as indicative, highlighting themes that deserve further investigation in other settings.

Interviewees were recruited as private individuals and gave informed consent to speak in a personal capacity. They did not speak on behalf of, nor represent, their employers or organisations, and no organisation approved or was asked to approve this manuscript. Organisations are named when referring to publicly available literature, or when they were specifically named within participants’ personal views, which are anonymised.

#### Interview procedures

Interviews were conducted via Microsoft Teams between July and August of 2023, lasting 50 to 65 min. Questions were developed from the literature review (see supplemental information), tailored for each group to guide discussions (see Table 2), though interviews were allowed to proceed organically following interviewees’ interests and concerns.

Questions followed a parallel structure, covering analogous themes to allow direct comparison between the groups, and were paired with fixed probes to address controversial issues directly. They aimed to (1) be participant-centred, covering personal motivations and experiences (e.g., joining the O&G industry or climate activism) to provide context, (2) address controversial issues directly, and (3) explore common ground and the usefulness of conversations to encourage participants to consider solutions and areas of agreement. Overall, they were designed to be open yet focused, providing enough guidance to keep discussions relevant while allowing participants full expression.

#### Transcription and data management

Interviews were recorded and transcribed manually by the first author, who had no prior relationships with any participant. Structured reflexivity procedures (e.g., field notes, positionality statements) were not undertaken. Data files were stored locally as encrypted Microsoft Word files, accessible only to the authors. Recordings of the interviews and full transcriptions will be retained and then destroyed in accordance with institutional guidelines.

#### Thematic analysis

Inductive thematic analysis was performed to identify areas of agreement and disagreement. This approach allowed for unbiased exploration and capture of unexpected and nuanced insights.^115^ Transcripts were analyzed to identify indications of agreement or disagreement between the groups, and similar codes were grouped together to form sub-themes of consensus and dissent. Sub-themes were evaluated repeatedly to ensure accurate representation of the data and to reflect the richness of interviewees’ perspectives.^116^

Coding was conducted manually by the first author in Microsoft Word. Double-coding was not undertaken. The codebook was iteratively developed during analysis and refined through repeated review of transcripts. Saturation was assessed qualitatively during coding; no new themes emerged after seven interviews.

#### Verification and rigor

Rigor was supported by systematic transcription and adherence to ethical approval conditions. Data triangulation was undertaken by comparing themes emerging from interviews with insights from publicly-available industry sources and academic literature (cited in the introduction and results).

## Supplemental information

Document S1. Note S1–S5
