# Supplementary material for: Consensus and dissent between climate activists and oil and gas employees in the United Kingdom
Source: iScience. 2025 Dec 17;29(1):114229. doi: 10.1016/j.isci.2025.114229 (PMC12834098; doi:10.1016/j.isci.2025.114229)
Supplement: Document S1. Note S1–S5 [file mmc1.pdf]

iScience, Volume *29*

Supplemental information

Consensus and dissent between climate activists and oil and gas employees in the United Kingdom

Ella Exley, Krista Halttunen, and Iain Staffell

Note S1: The energy transition

Interpretations of the term ‘energy transition’ have evolved, with contemporary discourse focusing on decarbonisation in the global energy system[1]. In this study, ‘energy transition’ refers to the ongoing shift from fossil fuels to renewable sources (such as wind and solar) to limit limiting global warming to 1.5°C, as per the 2015 Paris Agreement.

There is broad consensus among intergovernmental organisations, scientific bodies, and academics on the imperative to address climate change and accelerate the energy transition. The IPCC projects that by 2050, global coal, oil and gas usage must decline by 95%, 60% and 45%, respectively, from 2019 levels to align with 1.5°C pathways[2]. Energy scholars stress that a considerably faster energy transition is needed than historical, century-long transitions to mitigate climate change[1,3]. Current global policies fall short, with a 2.6–3.1°C rise projected by the end of the century[4].

Note S2: Decarbonisation within the O&G industry

Yergin[5] and Helm[6] highlight the roles of climate science and technological innovation in the steady decline of fossil fuels, requiring fossil fuel companies to adapt their traditional business models to remain viable. Shojaeddini et al. assess O&G companies’ adaptation efforts[7], evaluating strategies and investments of selected IOCs and NOCs. Building on Zhong and Bazilian[8], they propose criteria for evaluating companies’ efforts, observing that while IOCs are increasingly aligning with the energy transition, they remain overly cautious with their investments.

Beyond the strategies outlined by Shojaeddini et al.[7], some O&G companies are sharing proprietary data and research with policymakers, businesses and other stakeholders. Both BP[9] and Shell[10] project rapid declines in oil demand by 2040 due to increased vehicle efficiency and electrification[11]. This aligns with Helm’s assertion that O&G companies must transform to remain viable[6].

O&G workers face job insecurity and uncertainty in just transition policies, as renewable sectors might struggle to absorb displaced fossil fuel workers without policy intervention[12]. O&G trade unions face a 'jobs versus climate' tension, with some resisting rapid phase-out policies and others support emissions reductions[13]. Growing environmental concerns are also driving talent towards renewables, pressuring O&G firms to adapt and decarbonise[14]. However, "carbon lock-in" in higher education delays this transition, as energy professionals continue training in traditional sectors, creating a skills mismatch between industry needs and qualified labour[15].

While BP and Shell contribute to the energy transition literature, and Shojaeddini et al. indicate that steps towards decarbonisation by certain IOCs, the future of these companies remains controversial[7]. The O&G industry faces growing pressure to clarify its role in reducing GHG emissions reduction and climate change mitigation[16], as no majors' O&G production plans are currently aligned with 1.5°C[17]. BP and Shell initially committed to substantial oil production cuts by 2030, but scaled back these pledges in 2023, and are planning increased gas production in the coming years[18]. While clean energy investment is rising, O&G companies' contributions have been criticised as modest. Reclaim Finance[19] reported that in 2022, Europe's six largest energy majors allocated less than 25% of their total capital expenditure (CapEx) to low carbon projects (Figure 1), despite soaring revenues[18] and increased dividends[20] following higher energy prices. The IEA has stated the industry could play a larger role by applying its engineering, financial, and project-management expertise[21].

A large body of related literature explores the history of climate change denial in specific O&G companies, highlighting their efforts to protect traditional business models and raising uncertainty about their ability to transition. Banerjee et al.[22] uncovered internal documents showing that ExxonMobil scientists first warned executives about anthropogenic global warming in 1977. Further research suggests the American Petroleum Institute knew about global warming since the 1950s[23]; the coal industry since the 1960s[24]; General Motors and Ford since the 1970s[25]; and Shell since the 1980s[26]. Supran and Oreskes found ExxonMobil's advertorials overwhelmingly cast doubt on climate change, contradicting its internal reports and peer-reviewed papers authored by its employees[27]. Other studies document the gradual shift in companies' public discourse from overt

climate denial to subtle delaying tactics[28], such as redirecting responsibility for climate change mitigation onto individuals[29].

Note S3: A new wave of climate activism in the UK

On 31 October 2018, Extinction Rebellion (XR) emerged with a public declaration of rebellion against the UK government[30]. They demanded transparency on climate change, a commitment to net zero by 2025, and the creation of a citizens' assembly[31]. Founded by a small group of activists, XR aimed to revolutionise protest through non-violent mass civil disobedience and arrests[32]. Their strategy draws from Chenoweth's study of social movements[33], which highlights non-violence and civil disobedience for effective disruption and success. XR has expanded to 88 countries and inspired numerous spin-out groups[34], including Insulate Britain and Just Stop Oil (JSO). The key demand of JSO was for the UK government to stop licensing all new fossil fuel projects[35], which they declared as having been achieved, leading to their dissolution[36] in 2025. JSO is a focus within this study due to its large media coverage and divisive tactics, including slow marches, spray-painting structures, and disrupting major public events. JSO also explicitly targeted the fossil fuel industry as the principal contributor to climate change[37].

XR and JSO have faced much scrutiny. XR's roadblocks are seen by some as ineffective[38], and the group has been criticised as a faltering movement for failing to engage with politicians, with *'badly wrong'* tactics[39]. Media responses to JSO have been intense, branding the group as *'posh kids on vacation'*[40], and *'eco-zealots'*[41]. Kyyrö et al. note the tendency to *'religionise'* climate activists stems from activism's perceived incompatibility with economic rationality[42]. However, others credit XR with raising public awareness of environmental issues[43], and the disruptive demonstrations of JSO have been shown to increase public support for more moderate climate groups[44].

Note S4: Reconciling divergent perspectives

Kuhn highlights the growing relevance of reconciliation in today's world[45]. Amid complex challenges like the energy transition and increasing polarisation[46], facilitating reconciliation is increasingly important. While negotiation between activists and O&G employees is not the goal of this study, conflict resolution tools are used as an analytical framework to study the contrasting views of these groups.

Negotiation has attracted considerable attention within psychology[47]. Fisher et al. describe the difficulty of managing very separate interpretations of reality, shaped by individuals' personalities, values, emotions, and experiences[48]. To address this, we synthesise interview responses with existing literature to identify potential biases that influence disagreements between activists and O&G employees. Stone et al. emphasise avoiding blame and assuming intentions[49], while Malhotra stresses the importance of empathy[50]. This study therefore aims for neutrality in a contentious context and seeks depth in understanding interview responses.

Despite extensive study, practical integration of negotiation frameworks into research methodologies remains limited. Examples include analyses of perspectives on water issues in Phoenix, Arizona[51], and conflicts among stakeholders in Vietnamese tourism[52], both employing surveys.

Note S5: Illustrative quotes from the interviews

Consensus: The critical need to address climate change

'The tactics of climate denialism, then climate delay. Then, personal carbon footprints. These haven't been accidental. Like Shell, one of the pioneers of future scenarios I'm looking at...they raised their oil platforms to account for sea level rise before, whilst also denying that sea level rise is going to happen publicly.' (A1)

‘We’re literally burning. We’re literally cooking, cooking to death. So many, I mean hundreds of millions they say now, are being affected by the heat right now, today.’ (A2)

‘The whole concept of tipping points and runaway heating is the thing that's most scary to me and the thing that it's the thing that gives me a pit in my stomach and makes me absolutely terrified.’ (A3)

‘This is time limited. It's not like the longer we wait, it gets linearly worse. It gets exponentially worse.’ (A3)

‘There's articles about the tactics of the oil and gas industry and it's supposedly like they've moved away from climate denialism. That's no longer like a pushable rhetoric and that wouldn't make them look good. It’s all about appetite. That's actually not a technique that they're using anymore. And now it's like climate change and climate delay...Let's do individual personal carbon footprints, put it on the individual rather than on system change.’ (A3)

‘I think there's really rampant denialism within that industry.’ (A4)

‘I believe that climate change is extremely dangerous and action is urgent and far too little has been done in the last, I guess, well, you could say 30 years or more, but at least 20 years, by which time it became fairly clear about the nature of the risk and the time frames that were available to us and the sorts of major technological changes needed. So in the last 20 years, a huge amount has been done and achieved technologically, but the emission mitigation has been far too little. So I believe that, which I think they believe too, I believe in trying to stay within 1.5 degrees.’ (O1)

‘There is a consensus, it probably doesn’t feel like it, but there is a consensus that something needs to be done.’ (O2)

‘Honestly, I think it's so funny that they think that people are denying this. That's not what's happening. Not at all. No one's denying this at all. We know the numbers. In fact, it's very scary what the numbers are. And then I look and I go like, where are the solutions? And

I'm like crap. We need to work so much harder on this. And then I look to the really smart people within our company. I work with the really smart people in the industry, right? And they are really struggling to find the solutions for this. Right now, they are working hard to try and find solutions, but it's not easy. It's not easy. If we could do it, we would be doing it.' (O3)

'No one's a climate denier. Everyone thinks climate change is a big deal. Everyone thinks reaching net zero is a big deal.' (O4)

'So, I think people agree on the problem. Not all companies are the same, right? I mean, the one I know best is [O&G company], and that's at the more progressive end. And you've got companies like Exxon and Chevron at the less progressive end.' (OA1)

'I spoke at a couple of project conferences, and we did a poll, and in every poll in every place we did it, 99% of people said it was a problem apart from the US, where it was only two thirds. So it's quite a big difference in the US. And even that's amongst very well educated people. But I think they agree on the problem statements. They agree on the need for more renewables. They agree on the need for electric vehicles and the transition away from oil.' (OA1)

'They need to admit the truth of the climate crisis, which they're absolutely denying. You just talk to the people in the [O&G company] Scenarios team. I know people in that team, they are in denial.' (OA×)

Consensus: The importance of reducing energy demand

'I just have to keep in the present and do my best retrofitting this place I have and then learning to grow food, because that's where I see the future now, in community...It's taken me ten years to transition from where I was in my mindset, the capitalistic mindset with a good heart and all the rest, but I was still kind of sucked into the system.' (A2)

'I gave up flying in November 19, which meant most of my income disappeared, because I mostly worked for international companies and did projects. I stopped driving two years ago in the summer, I got rid of my car and I didn't use fossil fuels for the last two winters.

So, I’ve learned to live with cold and electric blankets and wood burner once in a while. I’m learning to live really simply. So, not buying things, only buying what I really need and thinking long term.’ (A2)

‘They're absolutely opposed to stopping new oil and gas because that's their business model.’ (A3)

‘If you want to transition, your quality of life will change. And how do you basically prepare people to get ready for that, rather than having, say, the COVID example, where it was like the pandemic is here and you must do this and then as soon as the rules lifted, everyone went back to normal. That’s not what you want. You want that these behaviours to stay and everybody accepts that this is the new way of life that may be there.’ (A4)

‘We should be reducing oil and gas usage very quickly, but stopping production before the demand has been reduced is problematic because we then won't be able to run society. So the focus should be on reducing demand by getting the electricity system less dependent and the transport system less dependent and heat less dependent. So we should be doing everything we can quickly to reduce demand and the reductions in production will quickly follow.’ (O1)

‘I think there is an element of there is still energy poverty in places in the world and affordable energy access is an issue. That said, we cannot deliver our Paris Agreement goals if everybody consumes energy the way that the West has over the last couple of decades. And so, I think it's a core thing that we need to address.’ (O2)

‘You're asking people to basically change the way that they live their lives, change the activities that they do in their lives, change their quality of life. These will all happen if you ask to change today, now. Like, right now. It's just what it is. It's just what it is. And so that's why I'm like, I agree we should do it. But how do we maintain this amazing quality of life that we've built up now while still transitioning? And I think the answer is you can't you have to give up something.’ (O3)

‘You have to reduce demand. Now, demand management is very, very unpopular and it’s certainly unpopular with the oil industry and it’s also unpopular with governments. But, that’s how we need to move forward. We need to have an acceptance that there will be changes in our lifestyle.’ (OA2)

Consensus: The central role of government and policy

‘The policies that we need are not in place, not in our country anyway, not in the UK. The UK is catastrophic. It’s a catastrophic situation. And, they’ve changed the law so you can’t protest anymore either. If you protest, you go straight to prison, more or less…I’m exaggerating…but, that’s what it is today. So, it’s becoming autocratic, the government to the system.’ (A2)

‘Fundamentally, the government are the only people that can step in to regulate this and do something. So the lens is not quite so much on the actual companies themselves because it's kind of inevitable if you don't have government policy that those companies are going to do the thing…they’re just going to operate from what’s economically beneficial to them.’ (A3)

‘London is the fossil fuel economic capital of the world or something, I don’t know what that means, but so much fossil fuel money goes through London. So, our government has a responsibility to stop this. They control the economy’ (A3)

‘[O&G company] complains a lot. They're very good at that. They complain a lot about the external regulation. They can only move as fast as the external government. True. But you can try and produce policies which the government can adopt. And I think there's a lot more that oil companies could do that would generate trust from the activists that they're actually trying to move to the right thing.’ (OA1)

‘I think the oil companies should be doing more. They ought to be lobbying themselves. They ought to be out lobbying for policies which reduce demand, and that would demonstrate that they are serious. If they were lobbying hard for things which would reduce gas demand, if they were lobbying for heat pump installation despite the fact they don't sell heat pumps, that would be a sign of good faith.’ (OA1)

‘The oil company doesn’t drive change, it follows change. This is the position that the Scenarios Group makes in [O&G company]. It’s that the oil industry meets the needs of society and it’s society that drives the oil industry. So, the oil industry is already admitting that it is a passive player…And yet, in private, they lobby and have lobbied for a very long time for no change or for slowing down change. So on the one hand, they say they’re a passive recipient of the economy and the demands of the public and industry for fuels, and on the other hand, they lobby for the continuation of fuels.’ (OA×)

‘People didn’t stop smoking when they were asked, they had to be legislated against. So the role of government is to legislate and they are failing to do that.’ (OA2)

Consensus: The continued use of existing UK O&G reserves

‘I think a lot of people think we're naive to the fact that if we just turn oil off, chaos would go down, which is definitely true. That's why the demand is like no new oil and gas, because if we wanted a wartime transition to renewables, which is what we're asking for, it's absolutely feasible. I think we've got an eight year reserve of oil in this country and if we treat this like the emergency it is, we can fully transition in that time. Wind and solar is nine times cheaper than oil and gas. But there's this idea that spouted that, no, it's too expensive, it would damage the economy. It's totally doable when you can think of the lives at stake’ (A3)

‘I haven't read their prospectus. So I'm aware of the headlines, of what they do, I see what they are doing in the news. I read the label: Just Stop Oil. And I do wonder what they mean behind that, specifically by when and in what ways.’ (O1)

‘I don't think it would be economically sensible to start exploring for new oil now in the knowledge in the UK that the exploration would be uncertain because most of the exploration has already been done. And taking, kind of what you just said, you'd spend a lot of money with some risk when you were exploring. And even after you find it, you've then got to think about how you develop it and what the cost will be. And it would be a fairly long term thing. I agree and anyone rationally looking at the economics I think would be disinclined to do that. But that's rather different from existing production infrastructure

where there are platforms and pipelines and there are known reservoirs nearby that may well have to be understood a bit more with some appraisal wells but are produceable relatively quickly and relatively cheaply. That would seem to me to make economic sense for the nation and sense from the point of view of security supply. And I think I'd say that it doesn't add to emissions because you'd be backing out oil that otherwise we would be importing from elsewhere. And indeed, the production of that oil and the transport of that oil from elsewhere, if you looked in net terms, the CO2 from the imported oil could potentially be higher than the CO2 from what was produced in the UK'. (O1)

'It's a nice idea to say we can just transition. It's easy, we can just move over, just give up the hydrocarbons, give up whatever, but the cost of living to do that is insanely high. It's not simple, and that's mainly to do with the fact that the scale of the technologies are not there.' (O3)

'Any sort of continued production of oil would probably be a loss to activists, and that's not something obviously the oil industry is going to agree to. They're not going to just turn off their fields.' (O4)

'The good thing about Just Stop Oil is it keeps it in the public eye. Bad thing is, I think it won't make any difference. If the North Sea oil and gas shut down tomorrow. It would make zero difference to the amount of oil and gas we use...and what it's really going to do is transfer revenue to governments like Russia and Saudi Arabia who are doing nothing about climate change...but most of them nevertheless instinctively feel that shutting oil production down will solve everything.' (OA1)

'The key demand of the International Energy Agency and the IPCC, which we are supporting rather than creating, is a transition out of oil and gas, which involves no new oil and gas...not stopping now. The rate at which fields will deplete will depend on the fields. So in the UK, we're probably looking with current rates of consumption at about eight years. The new licenses would add about four years to that, but of course would send a message to other countries that it's okay to do this...So the position of the IEA is about trade. The Climate Change Committee have said exactly the same thing, that we need to

stop new oil and gas production in this country. That sends a very important message to other countries.' (OA2)

Dissent: The scale and pace of change possible

'I think it sounds like to me it sounds like you're missing the point, if you're like, 'oh, but they're demanding net zero by 2025...but actually, I think that by 2030 would be far more realistic'. Kind of take it as what it is. It's a show of unrest and dissatisfaction with the the status quo, with respect to climate, with a lot of our energy transition...It's just, like, expressing discontent and I think you can read too much into it. When I was turning up there, it wasn't like '2025!'. I didn't have timelines in my head in any meaningful way. If Shell or something were like 'We hear your point, it's so valuable, but actually we're going to do 2027', that's not where the misalignment is. The misalignment is that they're still profiteering off oil and gas when it's causing mass harm around the world.' (A1)

'It feels like a technique. A technique of making groups more and more ineffective by getting bogged down in bureaucracy. It's supposedly quite an effective method of preventing action.' (A1)

'The Ukraine war is just a blessing to them. There's just no compassion in those industries. There's no compassion. It feels they're completely disconnected from humanity in the way they operate.' (A2)

'They're not providing affordable energy without extracting and exploiting fields. I mean, that's happening again in Nigeria...they're pretending to do good...whatever good they're doing is nothing in comparison to the amount of harm they're still causing.' (A2)

'These people are really important. They're advising governments, they're advising companies, and you can see how they'd been dragging their feet just for money. Just for money. It's all about money.' (A2)

'And these 1 billion refugees, how is that going to go for those people? That is literally like millions and millions of people dying and being forced from their land and it's just corporate greed that's driving it.' (A3)

‘I think it says a lot that Shell and BP and all of these are completely going back on their previous climate commitments just because they'd made a load of money because of energy prices. There's loads of complicated reasons for why they made money, but they made a load of money. And instead of investing it in clean energy, they have very much chosen the path that they want to take.’ (A4)

‘You either believe the IEA, which says no new fossil fuel investment post-2021, or you don’t.’ (A4)

‘I kind of see it as the line in the sand that a lot of climate activists have drawn, that’s what we’ve been told by the science, so that’s what we should be doing. Therefore, if the government isn’t acting on that or these companies aren’t acting on that, then they are in direct contravention with a 1.5 degree target...the oil and gas industry is actively choosing to not participate and accept the facts that have come out of several different entities now.’ (A4)

‘I think that response doesn't deny the fact that it's urgent or that we've only got a remaining budget of eight or ten years at present rates of emission. It's a question of what we can do about it and how quickly. So, the idea that we could stop emissions by 2025 or 2030 seems highly improbable.’ (O1)

‘I think between the adoption of the Paris agreement and about a year and a half ago, there was more alignment about trying to build that race to the top. I think over the last year that's deteriorated a little bit and I think that's, in part, because of the growing news that 1.5 is increasingly unlikely without overshoot. So, whereas before, there was more momentum and a mindset of ‘let's all do more’ to deliver Paris. Now, I think there's more question of like ‘what does it mean?’ and ‘what's realistic to ask of us?’...companies are like ‘but if it's not achievable, why are we expected to achieve it?’ And that's not a helpful conversation. So that is where we need a bridge.’ (O2)

‘I think applying their belief of what they saw six years ago isn't really fair because the world has changed and we simply don't have a choice. Again, I agree not enough is happening. But so I think that the combination of having a couple of people who are

hardcore activists who don't understand the energy space plus some people who have outdated knowledge is not necessarily always helpful.' (O2)

'Oil and gas companies have been making exorbitant amounts of money. There are institutional investors who don't care about climate, who just think that companies like ours should make as many returns as possible and provide that to them and they will invest in the transition in the meantime.' (O2)

'I do worry that there's a bias involved with there. They're definitely not up to date on the latest strategy. And I'm not saying that the latest strategy is the best, but it's still a huge improvement from when I joined, for sure.' (O3)

'At the end of the day, people have invested in us and we need to give them a return on their capital. That's just what a company is. That's not it's sole purpose, but it's one of the main things that they do.' (O3)

'I think the point of activism, right, is like maybe the demands that they are putting out are a bit unrealistic and I think that they might be aware of that, but it's sort of like a negotiation where you see extinction rebellion...they said they wanted net zero by 2025 and it's like that's just not a realistic thing that's going to happen. It would be lovely, but it's like there's no way you can decarbonize in like two years the entire world. So, I think I totally support the sentiment. I just think that maybe the purpose of climate activism is to be the unreasonable voice, almost, like to bring attention to the topic so that slightly more realistic goals can be met, if that makes sense.' (O4)

'But I think the question is people being unable to afford their energy bills is a much shorter term problem than climate change. It's a year to year issue. If people are so cold that people were dying because their houses were so cold a couple of winters ago, last winter with energy prices. So I think it's important to combat that just as of now. It has to be a balance, I would say. But at the end of the day, people have to be able to live.' (O4)

'I was having discussions with several activists…They're saying, 'Oh, nothing's changed'. And I said, '[O&G companies] are investing much more in recent years]. Isn't that a good

thing? And they're going to [continue increasing investments]’. But the perception is nothing's changed. And that's not really true. Things are changing.’ (OA1)

‘There are a whole bunch of practical constraints which are stopping the speed at which we do this rather than anything in principle.’ (OA1)

‘Investors are seeing that there's more money to be made in the oil and gas industry than there is in renewables.’ (OA1)

Oil companies are being encouraged to produce more, keep the prices down, which brings down inflation. So, there's kind of that side of it and that adds an additional tension as well. And, that’s why [an O&G company] backed away from its cut in production targets. That received backlash by the activists, but the share price went up 25%, and that’s the tension, right? That's, in many ways, the fundamental tension with trying to solve climate change in that somebody has to pay for decarbonisation. (OA×)

‘When I joined, [an O&G company] was a company talking about change. When I left, it was still a company just talking about change. There has been no change’ (OA2)

Dissent: The O&G industry’s place in the energy transition

‘They have such a track record of not holding the public interest at heart and just holding profits at heart that I think that kind of company shouldn’t hold the power that it has. I just don’t think they’re the good guys, and I think they’ve shown us enough evidence.’ (A1)

‘They’re becoming the enemy, they’re becoming our killers, literally. So, we’re talking war…some form of civil unrest or war or some form of resistance and protests against these companies.’ (A2)

‘Are these evil people? I’m quite close to that now, unfortunately. That’s judgemental, I don’t know them individually, so how can I describe them? But I am close to that now. It’s really hard to see the goodness.’ (A2)

‘The East African crude oil pipeline...Total is the majority shareholder...they’ve got this whole narrative about how they’re going to help Uganda develop and they’re helping the economy so much, even though they’re absolutely screwing over these people so directly. Not only are they displacing tens of thousands of people by building the pipeline without compensating them, but ten years down the line, when that oil is being burnt, those people can't grow food there. It's messed up! And it's all to get the super-rich in that country even richer. None of that is going to give any advantage to normal Ugandan people.’ (A3)

‘I think that those papers are a fundamental grounding to a lot of the reason why people want to dismantle the fossil fuel industry. Because if you look at that, it’s like, can these people change? Like, this is what they’ve been doing for the last 50 years.’ (A4)

‘As I imagine you are aware, the Committee on Climate Change has made clear that CCS is an essential technology for net zero. The Energy Transition Commission has produced a report with the same conclusion. It would have been much better if CCS had had such support for the last 20 years. It would have been possible, had CCS been deployed from the early 2000s, to have electricity systems that had largely been decarbonised by now, resulting in a lower atmospheric stock of CO2. Therefore, one of the suggestions I would like to make for consideration by [climate activist groups] is that they advocate CCS as a vital technology for net zero.’ (O1)

‘I can research as well. I don’t need you to help me go read up on the documents. I can do that myself. Where are the solutions? That’s really where 100% of the focus should be. All of this energy you have to scream around and stuff is like...now I’m getting annoyed because I’m trying to work on finding technologies. I’m working in engineering in the space to try and find solutions.’ (O3)

‘It's a fair point. I think it's super easy and not a totally wrong opinion to view the oil industry as the bad guys. I think if you look back...isn't there all that evidence that oil companies knew about climate change and were pretending they didn't?...Yeah, that's horrible. That's terrible. And if action had been taken at that time, I'm sure we would be a lot further along in the energy transition.’ (O4)

‘My thought was I wanted to...have a bit more influence and a bit more decision-making power...I wasn't necessarily supposed to be at an oil company...But I do think, and I'm sure loads of people disagree with this opinion...I think in five years, I'm going to have the potential to have a lot of ability to make good decisions and sometimes I'm glad that it's me working there and not someone who genuinely doesn't care.’ (O4)

‘It's much easier to say oil companies are bad bastards and it's all their fault. It's much easier to campaign that way than it is to say well, on balance they can be good but they've got to do X, Y and Z. That's a much more nuanced story but it's much easier to just say they're all bastards, should be shut down tomorrow.’ (OA1)

‘Extinction Rebellion in particular, they make a point not to propose any solutions...other than a sort of citizens assembly. They don't propose any solutions at all. And I personally find that frustrating because I want to get in and try and solve things’ (OA1)

‘If you’re the head of [O&G company], and you’re getting £6 million a year, you probably genuinely believe that you have a duty to provide energy to people and you know that if you did anything else, the company would probably get bought by someone else and you’d lose your job. You think you’re probably not that great, but you’re better than the other guy. It’s that little lawyer inside your head that’s justifying your behaviours...that’s what’s going on in the oil industry.’ (OA×)

‘I think that the oil and gas industry can only now change by accepting that it doesn’t have a role to play and it won’t do that.’ (OA2)

‘If that’s their position, then they’re not part of the solution. They’re the problem, because we have to stop using oil and gas. That’s a no brainer. That’s the basic position that we have to adopt, because 75% of the climate catastrophe that we’re in is due to the use of oil and gas.’ (OA2)

Supplemental References

1. Solomon, B. D. & Krishna, K. The coming sustainable energy transition: History, strategies, and outlook. *Energy Policy* **39**, 7422–7431 (2011).

2. IPCC. *Climate Change 2023: Synthesis Report. Contribution of Working Groups I, II and III to the Sixth Assessment Report of the Intergovernmental Panel on Climate Change*. https://www.ipcc.ch/report/ar6/syr/ (2023) doi:10.59327/IPCC/AR6-9789291691647.

3. Geels, F. W. Disruption and low-carbon system transformation: Progress and new challenges in socio-technical transitions research and the Multi-Level Perspective. *Energy Research & Social Science* **37**, 224–231 (2018).

4. UNEP. *Emissions Gap Report*. (United Nations Environment Programme, Nairobi, 2024). doi:10.59117/20.500.11822/46404.

5. Yergin, D. *The Quest: Energy, Security and the Remaking of the Modern World*. (Penguin Books, New York, 2012).

6. Helm, D. *Burn out: The Endgame for Fossil Fuels*. (Yale University Press, New Haven, 2017).

7. Shojaeddini, E., Naimoli, S., Ladislaw, S. & Bazilian, M. Oil and Gas Company Strategies Regarding the Energy Transition. *Progress in Energy* **1**, 012001 (2019).

8. Zhong, M. & Bazilian, M. D. Contours of the Energy Transition: Investment by International Oil and Gas Companies in Renewable Energy. *The Electricity Journal* **31**, 82–91 (2018).

9. BP. Net Zero Ambition Progress Update. https://www.bp.com/content/dam/bp/business-sites/en/global/corporate/pdfs/investors/bp-net-zero-progress-update-2023.pdf (2023).

10. Shell. The Energy Security Scenarios. https://www.shell.com/news-and-insights/scenarios/the-energy-security-scenarios.html (2023).

11. Halttunen, K., Slade, R. & Staffell, I. What if We Never Run Out of Oil? From Certainty of 'Peak Oil' to 'Peak Demand'. *Energy Research & Social Science* **85**, 102407 (2022).

12. Pai, S., Emmerling, J., Drouet, L., Zerriffi, H. & Jewell, J. Meeting well-below 2°C target would increase energy sector jobs globally. *One Earth* **4**, 1026–1036 (2021).

13. Normann, H. E. & Tellmann, S. M. Trade unions' interpretation of a just transition in a fossil fuel economy. *Environmental Innovation and Societal Transitions* **40**, 421–434 (2021).

14. Brannstrom, C., Ewers, M. & Schwarz, P. Will peak talent arrive before peak oil or peak demand?: Exploring whether career choices of highly skilled workers will accelerate the transition to renewable energy. *Energy Research & Social Science* **93**, 102834 (2022).

15. Vakulchuk, R. & Overland, I. The failure to decarbonize the global energy education system: Carbon lock-in and stranded skill sets. *Energy Research & Social Science* **110**, 103446 (2024).

16. Johnston, R., Blakemore, R. & Bell, R. The Role of Oil and Gas Companies in the Energy Transition. *Atlantic Council* https://www.atlanticcouncil.org/in-depth-research-reports/report/the-role-of-oil-and-gas-companies-in-the-energy-transition/ (2020).

17. Allen, T. & Coffin, M. *Paris Maligned: Why Investors Should Assess the Climate Alignment of Oil & Gas Companies*. (Carbon Tracker, 2022).

18. IEA. *World Energy Investment*. (International Energy Agency, Paris, 2023).

19. Reclaim Finance. Assessment of Oil and Gas Companies' Climate Strategy. https://reclaimfinance.org/site/en/assessment-of-oil-and-gas-companies-climate-strategy (2023).

20. Jolly, J. Shell drops target to cut oil production as CEO aims for higher profits. https://www.theguardian.com/business/2023/jun/14/shell-drops-target-to-cut-oil-production-as-ceo-guns-for-higher-profits (2023).

21. IEA. *The Oil and Gas Industry in Energy Transitions*. (International Energy Agency, Paris, 2020).

22. Banerjee, N., Song, L. & Hasemyer, D. Exxon's Own Research Confirmed Fossil Fuels' Role in Global Warming Decades Ago. https://insideclimatenews.org/news/16092015/exxons-own-research-confirmed-fossil-fuels-role-in-global-warming/ (2015).

23. Franta, B. Early Oil Industry Knowledge of CO_2 and Global Warming. *Nature Climate Change* **8**, 1024–1025 (2018).

24. Young, É. Coal Knew, Too. *Huffington Post* https://www.huffpost.com/entry/coal-industry-climate-change_n_5dd6bbebe4b0e29d7280984f (2019).

25. Joselow, M. GM, Ford Knew about Climate Change 50 Years Ago. *EE News* https://www.eenews.net/stories/1063717035 (2020).

26. Franta, B. Shell and Exxon's Secret 1980s Climate Change Warnings. *The Guardian* https://www.theguardian.com/environment/climate-consensus-97-per-cent/2018/sep/19/shell-and-exxons-secret-1980s-climate-change-warnings (2018).

27. Supran, G. & Oreskes, N. Assessing ExxonMobil's climate change communications (1977–2014). *Environ. Res. Lett.* **12**, 084019 (2017).

28. McKie, R. E. Climate Change Counter Movement neutralization techniques: A typology to examine the Climate Change Counter Movement. *Sociological Inquiry* **89**, 288–316 (2018).

29. Lamb, W. F. *et al.* Discourses of climate delay. *Glob. Sustain.* **3**, e17 (2020).

30. Wilson, T. & Walton, R. Extremism Rebellion: A Review of Ideology and Tactics. *Policy Exchange* https://policyexchange.org.uk/wp-content/uploads/2019/07/Extremism-Rebellion.pdf (2019).

31. Extinction Rebellion. Our Three Demands. https://extinctionrebellion.uk/about/ (2024).

32. Mansfield, F. Rebel for Life: Extinction Rebellion's Approach to the Climate Crisis. *Medicine, Conflict and Survival* **36**, 375–382 (2020).

33. Chenoweth, E. & Stephan, M. J. *Why Civil Resistance Works: The Strategic Logic of Nonviolent Conflict*. (Columbia University Press, New York, 2011).

34. Extinction Rebellion. XR in your area. https://rebellion.global/groups/#countries (2025).

35. Just Stop Oil. Background: Just Stop Oil. https://web.archive.org/web/20221023224405/https://juststopoil.org/background/ (2022).

36. Just Stop Oil. Just Getting Started. https://web.archive.org/web/20250627030540/https:/juststopoil.org/ (2025).

37. Gayle, D. 'It Was Terrifying': Stop Oil Activists on the New Battle Against Fossil Fuel. https://www.theguardian.com/environment/2022/oct/15/it-was-terrifying-stop-oil-activists-on-the-new-battle-against-fossil-fuel (2022).

38. Carlyle, G. XR: The 3.5 Percent Rule. https://peacenews.info/node/9359/xr-35-percent-rule (2019).

39. Spicer, A. The Extinction Rebels have got their tactics badly wrong. Here's why. https://www.theguardian.com/commentisfree/2019/apr/19/extinction-rebellion-climate-change-protests-london (2019).

40. Steafel, E. & Sigsworth, T. They're Now Seen as Posh Kids on Vacation': How Just Stop Oil Became a Laughing Stock. *The Telegraph* https://www.telegraph.co.uk/news/2023/07/27/just-stop-oil-prank-josh-archie-youtube/ (2023).

41. Dollimore, L. Just Stop Oil Throw Paint Over Energy Firm's Canary Wharf Office. *The Daily Mail* https://www.dailymail.co.uk/news/article-12237471/Just-Stop-Oil-activists-throw-orange-paint-energy-firms-Canary-Wharf-office.html (2023).

42. Kyyrö, J., Äystö, T. & Hjelm, T. The Cult of Greta Thunberg': De-legitimating Climate Activism with 'Religion'. *Critical Research on Religion* **11**, 133–149 (2023).

43. Smith, M. Concern for the Environment at Record Highs. *YouGov* https://yougov.co.uk/topics/politics/articles-reports/2019/06/05/concern-environment-record-highs (2019).

44. Ostarek, M., Simpson, B., Rogers, C. & Ozden, J. Radical climate protests linked to increases in public support for moderate organizations. *Nat Sustain* **7**, 1626–1632 (2024).

45. Kuhn, D. Why is Reconciling Divergent Views a Challenge? *Current Directions in Psychological Science* **29**, 27–32 (2019).

46. Newman, N., Fletcher, R., Robertson, C. T., Eddy, K. & Nielsen, R. K. *Digital News Report*. (Reuters Institute, Oxford, 2022).

47. Bazerman, M. H., Curhan, J. R., Moore, D. A. & Valley, K. L. Negotiation. *Annual Review of Psychology* **51**, 279–314 (2000).

48. Fisher, R., Ury, W. & Patton, B. *Getting to Yes: Negotiating Agreement without Giving In*. (Houghton Mifflin, Boston, 1991).

49. Stone, D., Patton, B. & Heen, S. *Difficult Conversations: How to Discuss What Matters Most*. (Portfolio Penguin, New York, 2011).

50. Malhotra, D. *Negotiating the Impossible*. (Berrett-Koehler Publishers, Oakland, California, 2016).

51. Larson, K. L., White, D. D., Gober, P., Harlan, S. & Wutich, A. Divergent Perspectives on Water Resource Sustainability in a Public–Policy–Science Context. *Environmental Science & Policy* **12**, 1012–1023 (2009).

52. Ngo, T., Hales, R. & Lohmann, G. Collaborative Marketing for the Sustainable Development of Community-based Tourism Enterprises: A Reconciliation of Diverse Perspectives. *Current Issues in Tourism* **22**, 2266–2283 (2018).
